# Cesium ion detection by terahertz light

**DOI:** 10.1038/s41598-017-08551-4

**Published:** 2017-08-24

**Authors:** Shin-ichi Ohkoshi, Marie Yoshikiyo, Asuka Namai, Kosuke Nakagawa, Kouji Chiba, Rei Fujiwara, Hiroko Tokoro

**Affiliations:** 1 Department of Chemistry, School of Science, The University of Tokyo, 7-3-1 Hongo, Bunkyo-ku Tokyo, 113-0033 Japan; 2 Cryogenic Research Center, The University of Tokyo, 2-11-16 Yayoi, Bunkyo-ku Tokyo, 113-0032 Japan; 3 Material Science Div., MOLSIS Inc., Tokyo Daia Bldg., 1-28-38 Shinkawa, Chuo-ku Tokyo, 104-0033 Japan; 4 Division of Materials Science, Faculty of Pure and Applied Sciences, University of Tsukuba, 1-1-1 Tennodai, Tsukuba Ibaraki, 305-8573 Japan

## Abstract

Recent developments in terahertz technologies provide new tools for analysis, inspection, and nondestructive sensing. If a heavy atom is encapsulated in a cage of a porous material, the atom should vibrate slowly and resonate with a low-frequency terahertz light. From this perspective, a cyanide-bridged metal framework is a suitable system because it contains many cages that can adsorb Cs ions. Herein we show the vibration mode of a Cs ion in a cage of a cyanide-bridged metal framework. First-principles phonon mode calculations and terahertz time-domain spectroscopy (THz-TDS) measurements indicate that the vibration mode of a Cs ion in a cyanide-bridged manganese-iron framework is at 1.5 THz, which is significantly apart from other lattice vibrations. Taking advantage of this feature, we develop a THz-light detection method for Cs ions, which is useful for non-contact sensing of Cs ions in dangerous environments or harmful circumstances.

## Introduction

Terahertz technologies are realizing innovative applications such as tests for illicit drugs in mail, quality management of medicine, semiconductor carrier density measurements, quality measurements of coating films, art conservation, and gas sensing at fire sites^1–6^. Terahertz technologies also provide fundamental information about the motions of free electrons, the rotation modes of molecules, the vibration modes of crystals, and precession of spins^7–17^. Recently, high-power terahertz sources and high-precision detectors have been developed to improve terahertz technologies. Additionally, high-resolution imaging using near-field imaging or terahertz tomography have been intensively studied^1–5, 18–20^. For example, field-enhancing arrays have been proposed for high-resolution imaging, enabling ultrasmall quantities of samples as small as tens of nanograms to be detected^21, 22^. Terahertz spectroscopy is a powerful tool. It can be used to measure various types of samples. For example, powder-form samples such as ceramics, proteins, and deoxyribonucleic acid have been measured using terahertz time-domain spectroscopy (THz-TDS)^23–26^.

**Figure 1:**
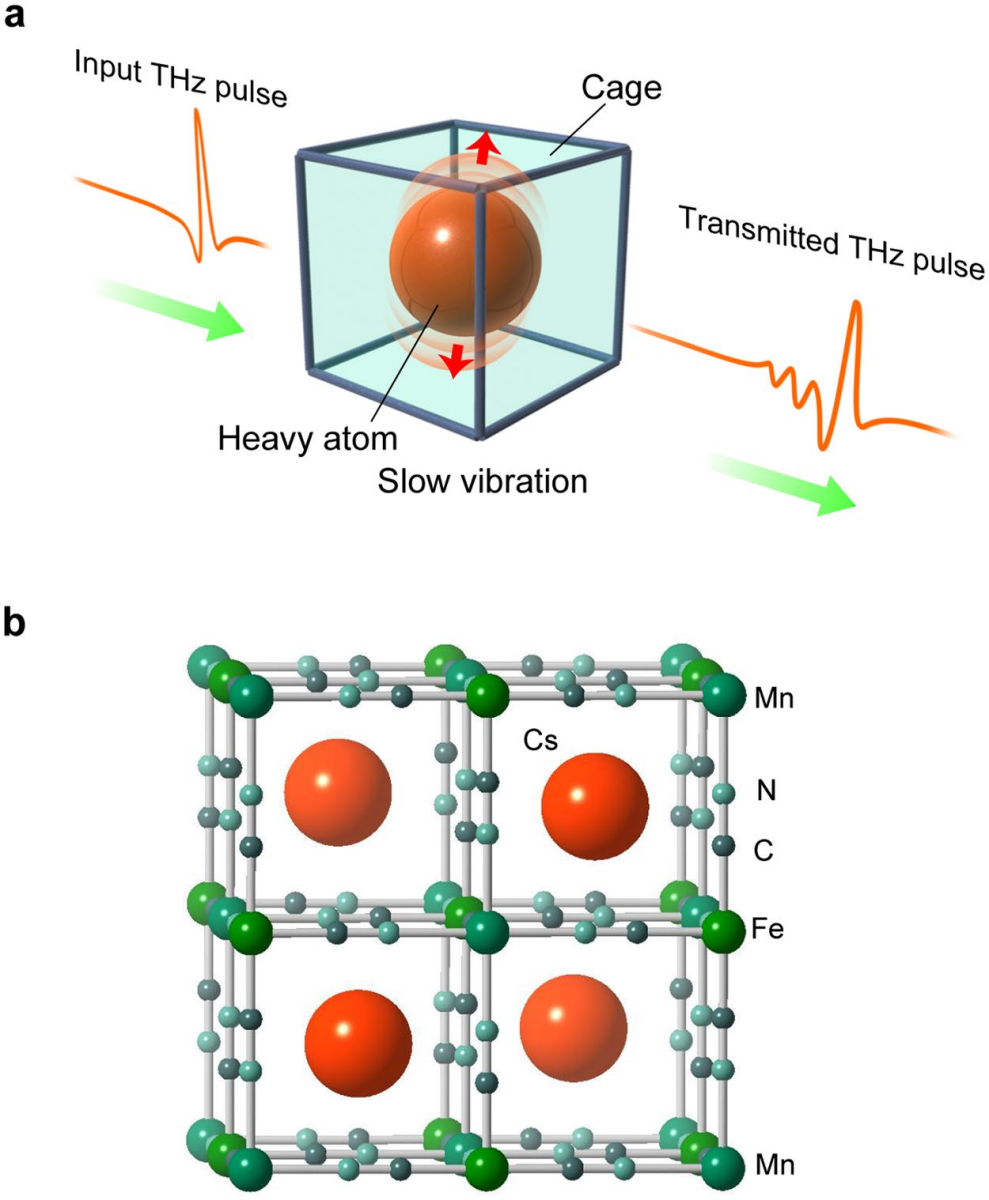
Concept of detecting the slow vibration of a heavy atom in a cage using THz-light. (**a**) Schematic illustration of a heavy atom encapsulated in a cage, which is expected to vibrate slowly and to resonate with low-frequency THz-light. This concept could be applied for non-contact sensing of heavy atoms. Input THz pulse (left) and transmitted THz pulse (right) are indicated with red lines. (**b**) Schematic crystal structure of the cesium-encapsulated cyanide-bridged manganese-iron framework. Red, blue green, green, dark gray, and light blue balls indicate Cs, Mn, Fe, C, and N atoms, respectively.

If a heavy atom is encapsulated in a cage of a porous material, the atom is expected to vibrate slowly and to resonate with a low-frequency terahertz light. To confirm this idea, metal frameworks such as metal-organic framework and cyanide-bridged metal framework are suitable systems because these are porous materials. Applications of these metal frameworks for organic molecule adsorbent, hydrogen adsorbent, and drug capsules are actively studied^27–30^. A cyanide-bridged metal framework has many cages in its three-dimensional network^31–39^, and the cage size matches the ionic radius of the Cs ion^40–43^. For example, a cyanide-bridged metal framework has been used as medicine and adsorbent for radioactive Cs ions. If a non-contact sensing technique can monitor the capture of a Cs ion by the metal framework, it would be very useful for the recovery operation of radioactive Cs ions in dangerous environments. From this viewpoint, we focus on THz technology because a heavy Cs atom should vibrate slowly in a cage and resonate with low-frequency THz-light (Fig. 1a and Movie S1). In the present work, we study the vibration mode (phonon mode) of a Cs ion in a cage of a cyanide-bridged metal framework using THz-spectroscopy. Additionally, we propose a THz-light detection method for Cs ions, which monitors how a Cs ion is captured by a cyanide-bridged metal framework. As a proof of concept, a cyanide-bridged manganese-iron framework is prepared.

## Results

### Cs cyanide-bridged metal framework

The sample was synthesized by reacting an aqueous solution of cesium chloride and manganese chloride with an aqueous solution of potassium ferricyanide. The chemical formula of the sample is Cs_0.90_Mn[Fe(CN)_6_]_0.93_·1.9H_2_O (**CsMnFe**), which has a cubic crystal structure (space group *F* $\overline{4}$3 *m*) with a lattice constant of *a* = 10.57986(13) Å (Fig. 1b, Fig. S1, and Table S1). The electronic structure of **CsMnFe** was investigated by periodic structure calculations using the Vienna *ab initio* Simulation Package program (Methods section, Fig. S2).

### Phonon mode calculations of the Cs vibration mode

**Figure 2:**
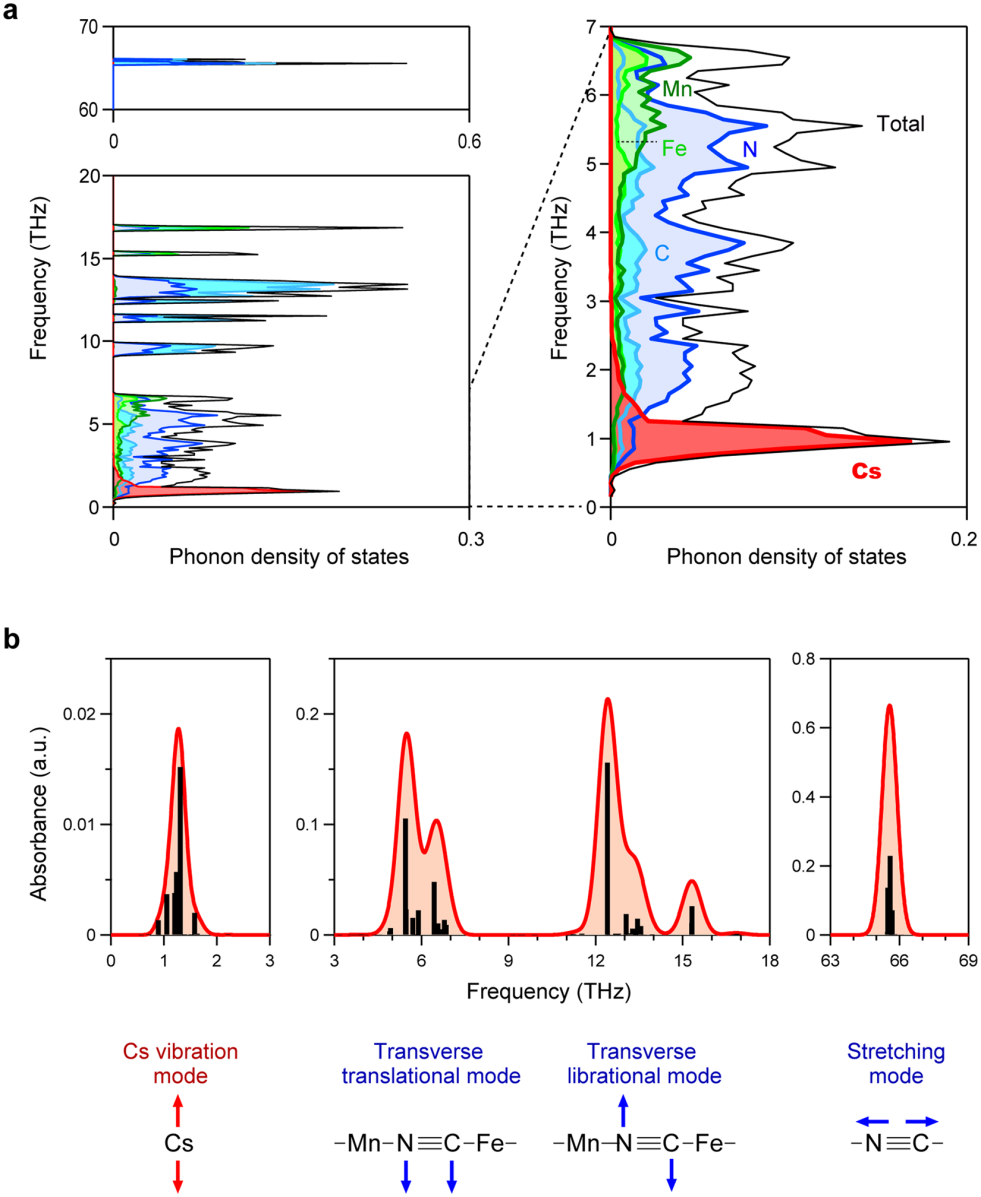
Phonon mode calculations of the vibration mode of Cs in a cyanide-bridged metal framework. (**a**) Phonon density of states of a Cs cyanide-bridged manganese-iron framework from first-principles phonon mode calculations (left). Enlarged view of the low frequency range of 0–7 THz (right). Black, red, green, light green, light blue, and blue lines indicate the total phonon density of states and the partial phonon density of states of Cs, Mn, Fe, C, and N, respectively. (**b**) Calculated optical transition probabilities of the phonon modes in the regions of 0–3 THz, 3–18 THz, and 63–69 THz. Black bars and red lines denote the transition probabilities and the calculated spectra considering the line width, respectively. Lower figure shows the atomic movements of the phonon modes at 1.3 THz, 5.5 THz, 12.4 THz, and 65.5 THz. Red and blue arrows indicate the movements of Cs and C≡N, respectively.

First-principles phonon mode calculations were performed using the Phonon code program (Methods section). Figure 2a and Figure S3 show the phonon density of states with an energy width of 1.0 meV (0.25 THz) and phonon dispersions, respectively. Figure 2b shows the calculated absorption spectra due to the transition probabilities of the IR active optical phonon modes considering absorption line width. In the low-frequency THz region, an absorption peak should be observed at 1.3 THz. The atomic movement of this phonon mode indicates that this absorption originates from the vibration modes of the Cs ion encapsulated in the cage of the cyanide-bridged metal framework (Fig. 2b and Supplementary Movie S2). In the frequency region of 3–18 THz, the vibration modes due to the Mn–N≡C–Fe transverse translational mode and transverse librational mode appear at 5.5 THz and 12.4 THz, respectively (Movies S3 and S4). Additionally, the C≡N stretching modes are at a distant high frequency around 65.5 THz (Movie S5).

### THz spectrum of Cs cyanide-bridged metal framework

**Figure 3:**
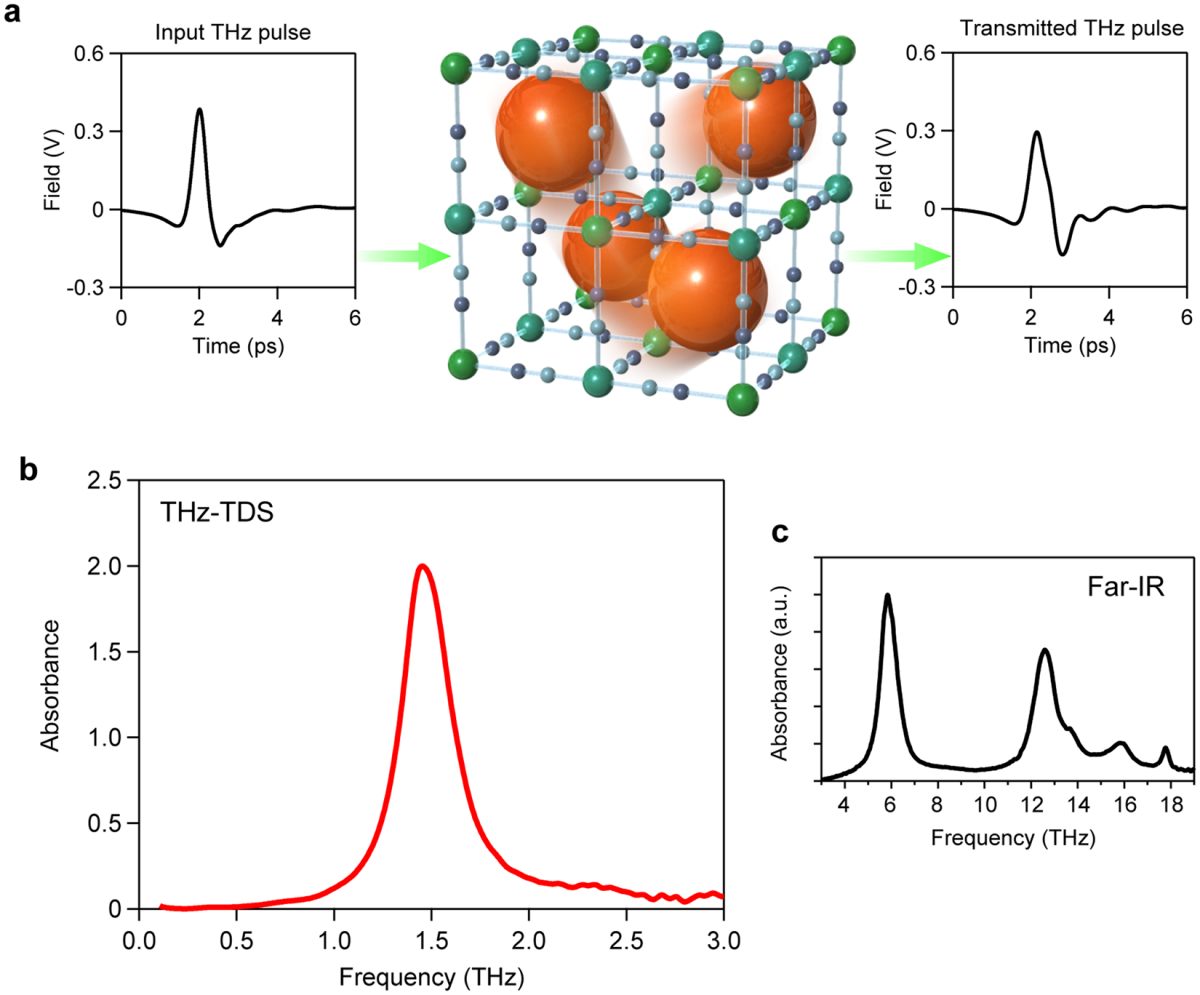
THz-TDS spectrum of **CsMnFe**. **(a**) Temporal waveforms of the input THz pulse (left) and transmitted THz pulse (right) for the THz-TDS measurement of the Cs vibration mode in the cyanide-bridged manganese-iron framework. (**b**) THz-TDS absorption spectrum of **CsMnFe**. (**c**) Absorption spectrum of **CsMnFe** measured by far-IR spectroscopy. Absorption peaks are assigned to the Mn–N≡C–Fe transverse translational or transverse librational modes.

Based on the theoretical calculations of the vibration modes of Cs, THz-TDS measurements of **CsMnFe** in the frequency region of 0–3 THz were performed. The temporal spectrum of the THz pulse wave was measured in the transmittance mode as shown in Fig. 3a (Methods section, Movie S6). A Fourier transformation of the temporal waveform yielded the frequency-dependent spectrum. The spectrum shows an absorption peak at 1.4 THz with a line-width of 0.3 THz (Fig. 3b). This absorption peak agrees well with the calculated Cs vibration mode, demonstrating that THz-TDS is useful to detect the Cs vibration mode in a cage. As for higher frequency vibration modes in the region of 3–19 THz, far-infrared measurements were performed; the observed spectrum is also consistent with the phonon mode calculations (Fig. 3c).

### The high Cs adsorption capacity monitored by THz light

**Figure 4:**
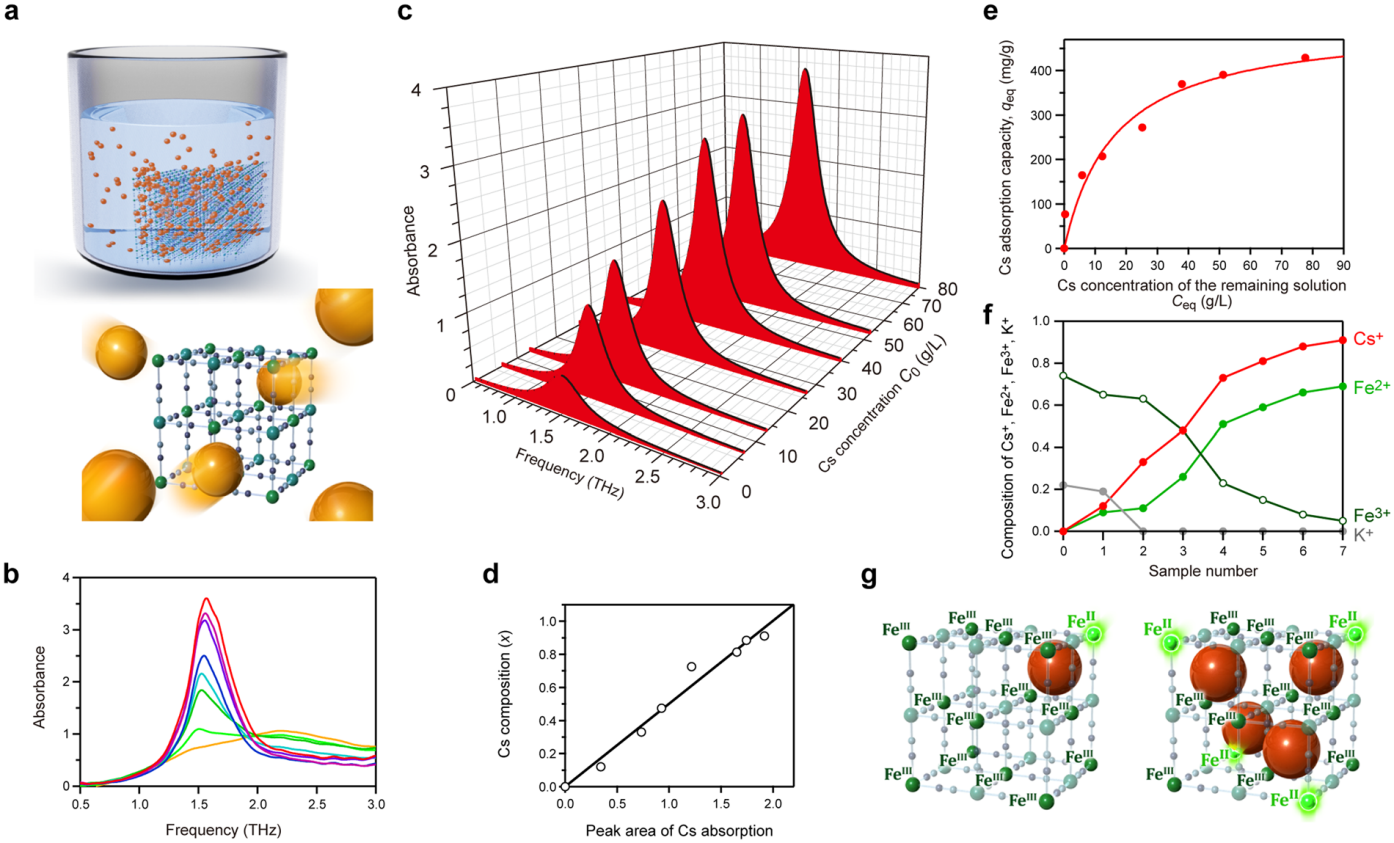
Highly efficient Cs adsorption of **MnFe** measured by THz light. **(a**) Schematic illustration of the cyanide-bridged metal framework adsorbing the Cs ions from the solution. **MnFe** (green framework) is immersed in a Cs ion solution as an adsorbent (upper left), and Cs ions (orange balls) are captured into the interstitial sites of the metal framework (lower right). (**b**) THz spectra of the samples recollected from Cs solutions of various concentrations. The peak intensity at 1.5 THz increases (orange → light green → green → light blue → blue → navy → purple → red) with the increase of Cs concentration (*C*_0_ = 0 → 79.7 g/L). (**c**) THz spectrum component due to the Cs vibration mode at 1.5 THz. (**d**) Cs composition (*x*) of the sample versus peak area for the samples recollected from various Cs solutions. Black line indicates the regression line. (**e**) *q*_eq_ of the samples recollected from the Cs solution versus the concentration of the remaining Cs solution at the equilibrium condition, *C*_eq_, derived from the *x* values. Red line shows the fitted curve following the Langmuir isotherm. The fitted curve shows a large *q*_max_ value of 511 mg/g. (**f**) Compositions of Cs^+^ (red), Fe^2+^ (light green), Fe^3+^ (green), and K^+^ (gray) for the samples after immersing in Cs solutions of various concentrations, *C*_0_ = 0 g/L (sample number 0), 0.665 g/L (1), 6.65 g/L (2), 13.3 g/L (3), 26.6 g/L (4), 39.9 g/L (5), 53.2 g/L (6), and 79.7 g/L (7). (**g**) The structures schematically showing the reduction of Fe ions from Fe^3+^ to Fe^2+^ by adsorption of Cs^+^. This is the origin of the highly efficient Cs adsorption performance of **MnFe**.

Taking advantage of such a feature, we investigated whether THz-TDS can monitor the cyanide-bridged metal framework capturing a Cs ion from a cesium ion containing aqueous solution (Fig. 4a and Movie S7). In this trial, a cyanide-bridged manganese-iron framework without cesium ions K_0.22_Mn[Fe(CN)_6_]_0.74_·4.3H_2_O (**MnFe**) was used as an adsorbent. The powder-form **MnFe** sample (0.25 g) was immersed in cesium chloride aqueous solutions (50 mL) with various concentrations (*C*_0_ = 0.665, 6.65, 13.3, 26.6, 39.9, 53.2, and 79.7 g/L). After 24 hours, the solution was filtered to collect the adsorbent sample.

The THz spectra of these precipitates were measured by THz-TDS using a polyethylene cell (Fig. S4). As a reference, the spectrum of the empty polyethylene cell was recorded. Figure 4b shows the obtained spectra. An absorption peak appears at 1.5 THz, which is due to the Cs vibration mode in the cage. Additionally, the weak broad peak at 2.2 THz is assigned to the water molecules inside the metal framework. The observed spectra were separated into these two components (Fig. S5). Figure 4c shows the absorption due to Cs ions at various Cs concentrations. Because each sample has a slightly different molar amount, the peak area of the Cs absorption was calibrated using the molar amount in the sample (See Methods). Figure 4d shows the Cs composition (*x*) of the chemical formula of the samples, Cs_*x*_Mn[Fe(CN)_6_]_0.74_·*z*H_2_O, plotted against the peak area of the Cs absorption. (The Methods section details the process to determine the chemical formulas and the peak area of Cs absorption.) The linear regression line corresponds to the conversion equation from the peak area of Cs absorption to the *x* values (Fig. 4d and Methods section). Using this conversion equation, the peak area of the Cs absorption for each sample can be converted to the *x* value. From the *x* values, the Cs adsorption capacity *q*_eq_ (mg/g) of the Mn–N≡C–Fe metal framework and the *C*_eq_ values can be obtained (Methods section). Figure 4e shows the *q*_eq_ versus *C*_eq_ plot. This plot is well fitted by the Langmuir equation, indicating that the saturated capability, *q*_max_, is 511 ± 55 mg/g. This *q*_max_ value is remarkably high compared to that of Prussian blue Fe[Fe(CN)_6_]_0.75_·*z*H_2_O adsorbent^44–48^. Figure 4f shows the metal ion compositions. The ratios of the metals (Cs, K, Mn, and Fe) were obtained by inductively coupled plasma mass spectroscopy. The amounts of C, H, and N contained in the samples were determined by standard microanalytical methods. The chemical formula, K^I^_*a*_Cs^I^_*b*_Mn^II^[Fe^II^(CN)_6_]_*c*_[Fe^III^(CN)_6_]_*d*_·*z*H_2_O, was determined considering charge neutrality (Methods section). Cs adsorption decreases Fe^3+^ and increases Fe^2+^. This is reasonable because the reduced valence state of the Fe ions in the metal framework is preferred in order to accept Cs^+^ ions. The reduction of Fe^3+^ to Fe^2+^ is the origin of the highly efficient Cs adsorption performance of the present system without collapsing of the framework (Fig. 4f,g) (Movie S8). In addition, the metal framework of **MnFe** has a high durability in an aqueous solution. Almost all of the adsorbent (average = 99%) can be recollected from the Cs ion solution.

Recently, metal frameworks have been studied by terahertz spectroscopy, and the vibration modes contributing to the lattice vibration of the framework have been reported^49^. In the present work, we clarified that a heavy atom such as a Cs ion vibrates slowly in the cage of a metal framework, and the Cs ion vibration mode lies at a low THz frequency. Although THz generation due to Cs ions has been known^50^, there has not been any observations of Cs ion by THz spectroscopy. Based on this feature, we demonstrate that the cyanide-bridged metal framework can be a marker to monitor the amount of Cs by combining it with THz-TDS measurements as well as an adsorbent to efficiently encapsulate Cs ions from cesium ion solutions. It should be noted that the absorption peaks of other alkali cations are observed at different frequencies because the vibration mode frequency depends on the type of alkali cation. Diffusion of radioactive Cs to the environment has been a serious issue, and its decontamination is an important mission after the accidents at nuclear power plants, e.g., the 2011 Fukushima accident^51, 52^. From this viewpoint, the combined system of THz-TDS with a cyanide-bridged metal framework demonstrated in this work will provide an effective detection method for non-contact sensing of Cs ions.

## Methods

### Electronic structure calculations

The electronic structure was calculated by first-principles calculations using the Vienna *ab initio* simulation package (VASP) program. The wave functions based on the plane waves and the potentials of the core orbitals were represented by the projector-augmented wave of Blöchl. The exchange-correlation term was evaluated by the generalized gradient approximation of Perdew, Burke, and Ernzerhof. The *U*–*J* value was set to 4.0 eV. The calculated density of states and band structure are shown in Fig. S2.

### Phonon mode calculations

The phonon mode was calculated based on the crystal structure of the cesium cyanide-bridged manganese-iron framework. The lattice parameters and atomic positions were calculated with an energy cut-off of 500 eV and 3 × 3 × 3 *k* mesh until satisfying a 10^−5^ eV pm^−1^ force tolerance. $\sqrt{2}\times \sqrt{2}\times 1$ supercell of the optimized structure containing four Cs ions alternatively in the interstitial sites was used for the phonon mode calculations by the direct method implemented in Phonon code with displacements of 2 pm.

### THz-TDS measurements

THz spectra were measured by a THz-TDS system of Advantest tas7500su, equipped with a Cherenkov type THz generator and emitter using a LiNbO_3_ waveguide and a 1550-nm fiber laser (50 fs, 150 mW) (Fig. S4). The THz pulse was condensed with a Si lens and paraboloidal mirrors and was irradiated into the sample, which was placed in a polyethylene cell. The electric fields of the transmitted THz pulse wave formed in the time domain were obtained, and the power spectrum was obtained by Fourier transformation. Absorbance was obtained from the power spectra of the reference and the sample.

### Cs adsorption experiment

In the adsorption experiment, the cyanide-bridged manganese-iron framework without cesium ions **MnFe** was used as an adsorbent. The sample was prepared by reacting an aqueous solution of manganese chloride (0.1 mol L^−1^) with an aqueous solution of potassium ferricyanide (0.1 mol L^−1^), and its formula was K_0.22_Mn[Fe(CN)_6_]_0.74_·4.3H_2_O. The powder-form **MnFe** sample was immersed in a cesium chloride aqueous solution at various concentrations and then filtered. Their formulas were investigated by elemental analyses. The formulas of the collected samples after immersing were as follows: Cs_0.12_K_0.19_Mn[Fe(CN)_6_]_0.74_·*z*H_2_O, Cs_0.33_Mn[Fe(CN)_6_]_0.74_·*z*H_2_O, Cs_0.48_Mn[Fe(CN)_6_]_0.74_·*z*H_2_O, Cs_0.73_Mn[Fe(CN)_6_]_0.74_·*z*H_2_O, Cs_0.81_Mn[Fe(CN)_6_]_0.74_·*z*H_2_O, Cs_0.88_Mn[Fe(CN)_6_]_0.74_·*z*H_2_O, and Cs_0.91_Mn[Fe(CN)_6_]_0.74_·*z*H_2_O. The Supplementary information contains the results of elemental analyses. In the THz-TDS measurement, the peak intensity at 1.5 THz increases with the increase of composition of Cs ions in the formula, and the broad peak at 2.2 THz simultaneously disappears. The THz spectra were separated into the vibration modes at 1.5 and 2.2 THz. Because the molar amounts of the samples slightly differ (Fig. S4, caption), the absorption intensity (peak area) was calibrated with the mole number of the sample. The mole number of the THz-TDS–measured sample which had been collected from the cesium chloride aqueous solution of *C*_0_ = 6.65 g/L (40.6 µmol) was used as a reference. The Cs composition (*x*) was plotted against the normalised peak area of the Cs absorption. The composition (*x*) is well fitted by a first-order regression function with a slope of 0.501. Hence, this relation was used as the conversion equation to transfer the peak area of the Cs absorption of the THz spectrum to the Cs composition for a collected sample.

### Material characterization and physical property measurements

The chemical formulas of the samples were determined by elemental analyses. The ratios of the metals (Cs, K, Mn, and Fe) were obtained by inductively coupled plasma mass spectroscopy using Agilent 7700. The amounts of C, H, and N contained in the samples were determined by standard microanalytical methods. The chemical formula, K^I^_*a*_Cs^I^_*b*_Mn^II^[Fe^II^(CN)_6_]_*c*_[Fe^III^(CN)_6_]_*d*_·*z*H_2_O, was determined considering charge neutrality. Because the valences of K^I^, Cs^I^, Mn^II^, [Fe^II^(CN)_6_], and [Fe^III^(CN)_6_] are +1, +1, +2, −4, and −3, respectively, *a* + *b* + 2 − 4*c* − 3*d* = 0 should be satisfied. For the far-infrared measurements, a JASCO 6100 spectrometer was used. XRD measurements were performed with a Rigaku Ultima IV diffractometer with a Cu *K*_*α*_ radiation (*λ* = 1.5418 Å). The PDXL program of Rigaku was used for Rietveld analyses.

### Adsorption analysis using Langmuir model

Using the *x* values obtained from the THz spectra by the conversion equation, the *q*_eq_ and *C*_eq_ values are calculated by ${q}_{{\rm{eq}}}=({M}_{{\rm{Cs}}}/{M}_{{\rm{MnFe}}})x$ and${C}_{eq}={C}_{{\rm{0}}}-$ $(m{M}_{{\rm{Cs}}}/V{M}_{{\rm{MnFe}}})x$, where *M*_Cs_ and *M*_MnFe_ are the atomic weight of Cs and the formula weight of the adsorbent, respectively, *m* is the sample weight of the immersed adsorbent, and *V* is the volume of the Cs ion solution. The *q*_eq_ versus *C*_eq_ plot was fitted by the Langmuir model expressed by ${q}_{{\rm{eq}}}={q}_{{\rm{\max }}}a{C}_{{\rm{eq}}}/(1+a{C}_{{\rm{eq}}})$, where *a* is a constant. The THz-TDS measurement system has a dynamic range of 60 dB, and the measurement is possible up to Cs saturated solution under the present condition. The lower limit of the detection sensitivity is several ppm.

## Electronic supplementary material

Supplementary Information

Supplementary Movie S1

Supplementary Movie S2

Supplementary Movie S3

Supplementary Movie S4

Supplementary Movie S5

Supplementary Movie S6

Supplementary Movie S7

Supplementary Movie S8
