# Supplementary material for: Cesium ion detection by terahertz light
Source: Sci Rep. 2017 Aug 24;7:8088. doi: 10.1038/s41598-017-08551-4 (PMC5570936; doi:10.1038/s41598-017-08551-4)
Supplement: Supplementary file 1 — Supplementary Information [file 41598_2017_8551_MOESM1_ESM.pdf]

Cesium ion detection by terahertz light

Shin-ichi Ohkoshi[1,2,*], Marie Yoshikiyo,[1] Asuka Namai,[1] Kosuke Nakagawa,[1] Kouji Chiba,[3]
Rei Fujiwara,[4] and Hiroko Tokoro[1,4,*]

[1]*Department of Chemistry, School of Science, The University of Tokyo*
7-3-1 Hongo, Bunkyo-ku, Tokyo 113-0033, Japan
[2]*Cryogenic Research Center, The University of Tokyo,*
2-11-16 Yayoi, Bunkyo-ku, Tokyo 113-0032, Japan
[3]*Material Science Div., MOLSIS Inc.,*
Tokyo Daia Bldg., 1-28-38 Shinkawa, Chuo-ku, Tokyo 104-0033, Japan
[4]*Division of Materials Science, Faculty of Pure and Applied Sciences, University of Tsukuba*
1-1-1 Tennodai, Tsukuba, Ibaraki 305-8577, Japan

*Correspondence should be addressed to S. O. and H. T.
ohkoshi@chem.s.u-tokyo.ac.jp, tokoro@ims.tsukuba.ac.jp

Contents: **Page**

1. Legends for Supplementary Movies S1–S8. .... S2–S3

2. Synthesis and crystal structure of **CsMnFe**. .... Fig. S1, Table S1 S4

3. First-principles electronic structure calculations. .... Fig. S2 S5

4. First-principles phonon mode calculations. .... Fig. S3 S6

5. THz-TDS measurements. .... Figs. S4 and S5 S7–S8

6. Elemental analyses of the samples recollected from Cs solutions. S9

§ 1. Legends for Supplementary Movies 1–8.

Supplementary Movie S1: The concept of non-contact sensing of heavy atoms using THz-light. Schematic illustration of a heavy atom encapsulated in a cage is initially shown. The heavy atom in the cage vibrates slowly and resonate with a low frequency THz light. This phenomenon can be applied as effective detection method for non-contact sensing of heavy atoms such as Cs.

Supplementary Movie S2: Calculated phonon mode of the Cs vibration in the cyanide-bridged metal framework. The crystal structure of the cyanide-bridged manganese-iron framework is initially shown. Turquoise, green, navy, and light blue spheres represent Mn, Fe, C, and N atoms respectively. Then Cs ions (orange spheres) are adsorbed at the interstitial sites of the framework and vibrate in the cages, displaying the atomic movements of the calculated phonon mode at 1.3 THz.

Supplementary Movie S3: Calculated phonon mode of the transverse translational mode of the Mn–N≡C–Fe framework. This movie shows the atomic movement of the phonon mode at 5.5 THz assigned to the transverse translational mode of the Mn–N≡C–Fe framework. Orange, turquoise, green, navy, and light blue spheres denote Cs, Mn, Fe, C, and N atoms, respectively.

Supplementary Movie S4: Calculated phonon mode of the transverse librational mode of the Mn–N≡C–Fe framework. This movie shows the atomic movement of the phonon mode at 12.4 THz assigned to the transverse librational mode of the Mn–N≡C–Fe framework. Orange, turquoise, green, navy, and light blue spheres indicate Cs, Mn, Fe, C, and N atoms, respectively.

Supplementary Movie S5: Calculated phonon mode of the C≡N stretching mode of the cyanide-bridged metal framework. This movie shows the atomic movement of the phonon mode at 65.6 THz assigned to the C≡N stretching mode of the manganese-iron metal framework. Orange, turquoise, green, navy, and light blue spheres represent Cs, Mn, Fe, C, and N atoms, respectively.

Supplementary Movie S6: THz-TDS measurement system for detection of the Cs vibration mode. A diagram of the THz-TDS measurement system is initially shown. THz pulse is irradiated into the sample, and the transmitted THz pulse is detected in the time domain to obtain a temporal wave form. The irradiated and transmitted temporal waveforms are Fourier transformed to obtain the absorption spectrum.

Supplementary Movie S7: Cesium ion adsorption by cyanide-bridged metal framework. In this movie, a schematic illustration of the cyanide-bridged metal framework capturing the Cs ions (orange spheres) from a cesium ion containing solution is shown. The amount of Cs adsorbed in the metal framework can be monitored by THz light (orange lines).

Supplementary Movie S8: Mechanism for the high Cs adsorption capacity of the cyanide-bridged manganese-iron framework. When Cs^{+} ion is adsorbed to cyanide-bridged manganese-iron framework, the Fe^{3+} is reduced to Fe^{2+} to maintaining charge neutrality of the framework.

§ 2. Synthesis and crystal structure of CsMnFe.

CsMnFe was prepared by reacting an aqueous solution of cesium chloride (0.3 mol dm^{-3}) and manganese chloride (0.1 mol dm^{-3}) with an aqueous solution of potassium ferricyanide (0.1 mol dm^{-3}) according to the modified method based on our previous report (*Dalton Trans.* 5046 (2006)). Elemental analyses showed that the formula of **CsMnFe** is $Cs_{0.90}Mn[Fe(CN)_6]_{0.93}\cdot 1.9H_2O$. Calculated: Cs, 29.50; Mn, 13.52; Fe, 12.78; C, 16.49; N, 19.23%; Found: Cs, 29.46; Mn, 13.50; Fe, 12.24; C, 16.53; N, 19.35 %.

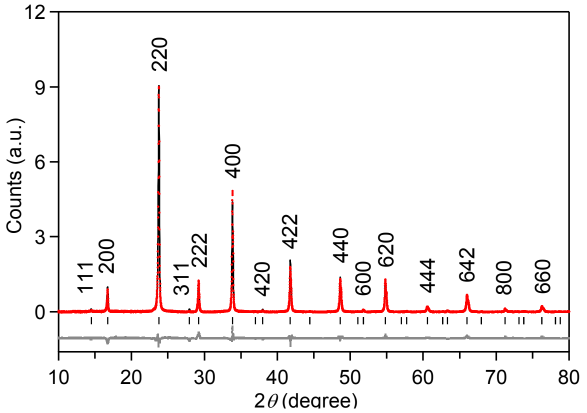

Figure S1. XRD patterns and Rietveld analyses of **CsMnFe**. Red dots are the experimentally observed XRD pattern. Black line is the calculated XRD pattern obtained by Rietveld analysis. Gray dots are the differences between the observed and calculated XRD patterns. Black bars represent the calculated positions of the Bragg reflections of cubic structure ($F\bar{4}3m$). Each diffraction peak is labeled with mirror indices showing the crystallographic direction.

Table S1. Atomic positions and occupancies of **CsMnFe** obtained by Rietveld refinement of the XRD pattern.* Positions of the constituent atoms of **CsMnFe** are indicated. Mn position is taken as the origin. *x*, *y*, and *z* are corresponding to the crystallographic *a*-, *b*-, and *c*-axes.

Atoms	*x*/*a*	*y*/*b*	*z*/*c*	Occupancy
Cs1	0.2500	0.2500	0.2500	0.4900
Cs2	0.7500	0.7500	0.7500	0.4100
Mn	0.0000	0.0000	0.0000	1.0000
Fe	0.5000	0.5000	0.5000	0.9300
C	0.0000	0.0000	0.3174	0.9300
N	0.0000	0.0000	0.2068	0.9300
O1	0.0000	0.0000	0.2068	0.0700
O2	0.2523	0.2523	0.2523	0.1110
O3	0.6381	0.6381	0.6381	0.1090

*Crystal system: cubic, space group; $F\bar{4}3m$, lattice parameter: $a = b = c = 10.57986(13)$ Å, $Z = 4$.

§ 3. First-principles electronic structure calculations.

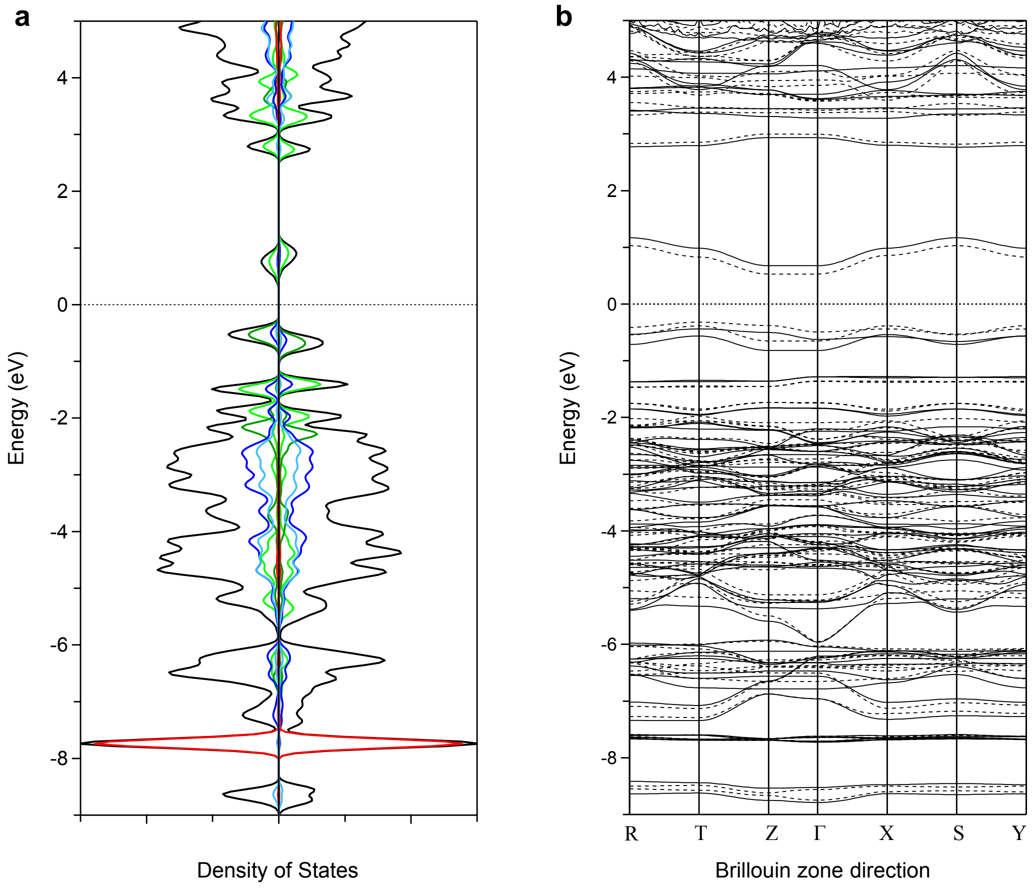

Figure S2. Electronic structure of cesium-encapsulated cyanide-bridged manganese-iron framework obtained by first-principles calculations using the VASP program. **a**, Total density of states (black line) and partial density of states of Cs (red line), Mn (green line), Fe (light green line), C (light blue line), and N (blue line). **b**, Band structure showing the energy states of up-spin (solid lines) and down-spin (dotted lines).

§ 4. First-principles phonon mode calculations.

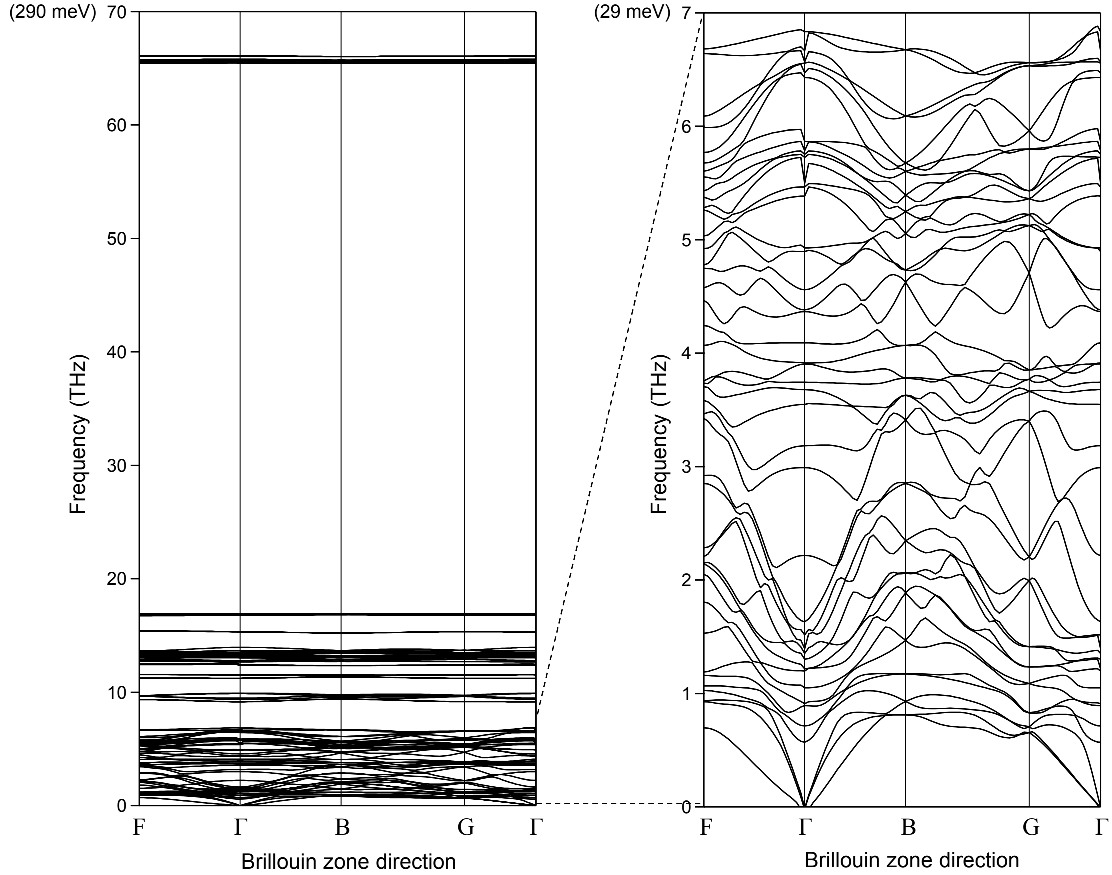

Figure S3. Phonon dispersion of the cesium-encapsulated cyanide-bridged manganese-iron framework obtained by first-principles phonon mode calculations. Left shows the full frequency range of 0–70 THz (0–290 meV), and right is an enlargement in the range of 0–7 THz (0–29 meV).

§ 5. THz-TDS measurements.

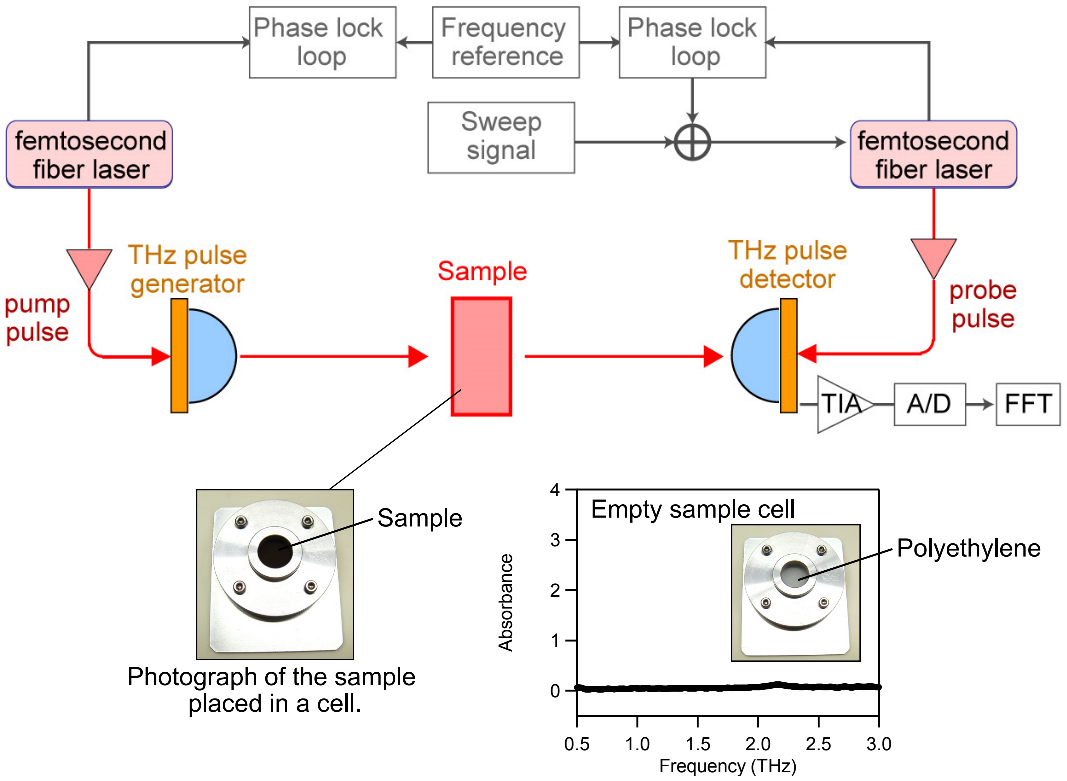

Figure S4. Schematic diagram of the THz-TDS measurement system. Powder-form samples were placed in a polyethylene cell. Lower right figure shows the reference spectrum of the empty polyethylene cell. The amount of the framework was adjusted to a constant value based on the amount of samples used for the measurement and the chemical formula, and the amount of Cs in the framework was monitored. In the experiments for the high Cs adsorption capacity monitored by THz light, the sample amounts placed into the polyethylene cell for the THz-TDS measurement are as follows: 13.5 mg (43.9 μmol), 13.4 mg (40.6 μmol), 13.9 mg (41.2 μmol), 14.2 mg (40.3 μmol), 14.2 mg (39.4 μmol), 14.5 mg (39.8 μmol), and 17.4 mg (47.4 μmol) for C_0 = 0.665, 6.65, 13.3, 26.6, 39.9, 53.2, and 79.7 g/L, respectively.

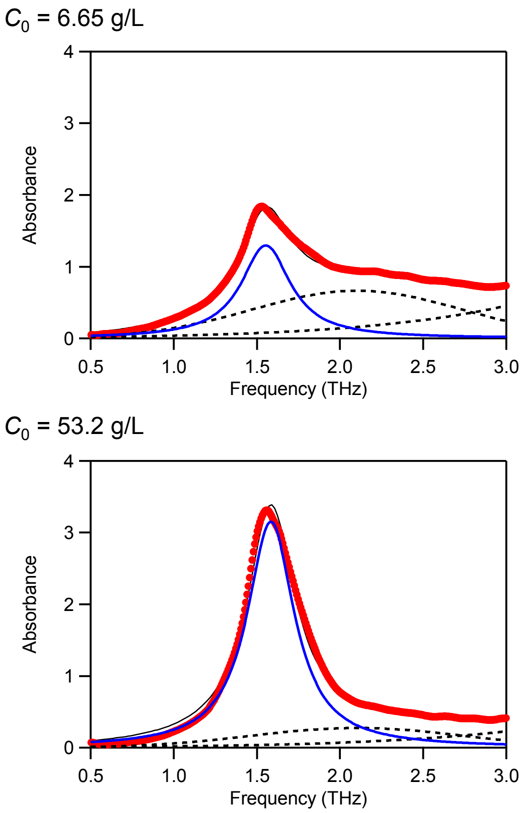

Figure S5. Spectral waveform separation. Spectral waveform separation of the spectra for C_0 = 6.65 g/L and 53.2 g/L are shown as examples. Red marks and the solid black lines represent the observed spectra and the calculated total spectra, respectively. Solid blue lines and black dotted lines show the contributions of the vibration mode of the Cs ion and the water molecules, respectively.

§ 6. Elemental analyses of the samples recollected from Cs solutions.

The elemental analyses results of **MnFe** and the samples recollected from various Cs concentration solutions are as follows: $K_{0.22}Mn[Fe(CN)_6]_{0.74}\cdot 4.3H_2O$, Calculated: K, 2.89; Mn, 18.44; Fe, 13.87; C, 17.90; N, 20.88; H, 2.91%; Found: K, 3.33; Mn, 18.87; Fe, 13.86; C, 18.07; N, 21.13; H, 3.30%. $Cs_{0.12}K_{0.19}Mn[Fe(CN)_6]_{0.74}\cdot 4.0H_2O$, Calculated: Cs, 5.19; K, 2.42; Mn, 17.88; Fe, 13.45; C, 17.36; N, 20.25%; H, 2.62%; Found: Cs, 5.27; K, 2.40; Mn, 17.88; Fe, 13.36; C, 17.71; N, 20.73; H, 2.56% (C_0 = 0.665 g/L). $Cs_{0.33}Mn[Fe(CN)_6]_{0.74}\cdot 4.1H_2O$, Calculated: Cs, 13.31; Mn, 16.67; Fe, 12.54; C, 16.18; N, 18.88; H, 2.51%; Found: Cs, 13.00; Mn, 16.27; Fe, 12.13; C, 16.93; N, 19.89; H, 2.01% (6.65 g/L). $Cs_{0.48}Mn[Fe(CN)_6]_{0.74}\cdot 3.4H_2O$, Calculated: Cs, 18.94 Mn, 16.31; Fe, 12.27; C, 15.83; N, 18.47; H, 2.03%; Found: Cs, 18.67; Mn, 16.01; Fe, 12.09; C, 16.38; N, 19.25; H, 1.68% (13.3 g/L). $Cs_{0.73}Mn[Fe(CN)_6]_{0.74}\cdot 2.4H_2O$, Calculated: Cs, 27.56; Mn, 15.61; Fe, 11.74; C, 15.15; N, 17.67; H, 1.37%; Found: Cs, 27.34; Mn, 15.33; Fe, 11.77; C, 15.43; N, 18.09; H, 1.10% (26.6 g/L). $Cs_{0.81}Mn[Fe(CN)_6]_{0.74}\cdot 2.3H_2O$, Calculated: Cs, 29.83; Mn, 15.22; Fe, 11.45; C, 14.78; N, 17.24; H, 1.28%; Found: Cs, 30.02; Mn, 15.16; Fe, 11.37; C, 15.08; N, 17.70; H, 0.98% (39.9 g/L). $Cs_{0.88}Mn[Fe(CN)_6]_{0.74}\cdot 2.0H_2O$, Calculated: Cs, 32.06; Mn, 15.06; Fe, 11.33; C, 14.62; N, 17.05; H, 1.11%; Found: Cs, 32.23; Mn, 14.98; Fe, 11.45; C, 14.74; N, 17.27; H, 0.89% (53.2 g/L). $Cs_{0.91}Mn[Fe(CN)_6]_{0.74}\cdot 1.9H_2O$, Calculated: Cs, 32.96; Mn, 14.97; Fe, 11.26; C, 14.53; N, 16.95; H, 1.04%; Found: Cs, 32.84; Mn, 14.89; Fe, 11.22; C, 14.54; N, 17.07; H, 0.86% (79.7 g/L).
